# Mapping of resistance to corn borers in a MAGIC population of maize

**DOI:** 10.1186/s12870-019-2052-z

**Published:** 2019-10-17

**Authors:** José Cruz Jiménez-Galindo, Rosa Ana Malvar, Ana Butrón, Rogelio Santiago, Luis Fernando Samayoa, Marlon Caicedo, Bernardo Ordás

**Affiliations:** 1 Misión Biológica de Galicia, Spanish National Research Council (CSIC), Apartado 28, 36080 Pontevedra, Spain; 2 National Institute of Forestry, Agriculture and Livestock Research (INIFAP), Ave. Hidalgo 1213, Cd. Cuauhtémoc, 31500 Chihuahua, Mexico; 3 Departamento Biología Vegetal y Ciencias del Suelo, Unidad Asociada BVE1-UVIGO y MBG (CSIC), Facultad de Biología, Universidad de Vigo, Campus As Lagoas Marcosende, 36310 Vigo, Spain; 4 North Carolina State University, 4210 Williams Hall 101, Derieux Place, Raleigh, NC 27695 USA; 5 Department of Crop and Soil Sciences, North Carolina State University, Raleigh, NC 27695-7620 USA; 6 Instituto Nacional de Investigaciones Agropecuarias (INIAP), 170315 Quito, Ecuador

**Keywords:** Maize, Mediterranean corn borer, *Sesamia nonagrioides*, Resistance, Mapping with multi-parent advanced generation InterCrosses (MAGIC) populations, Quantitative trait loci (QTL), Genome wide association analysis (GWAS)

## Abstract

**Background:**

Corn borers constitute an important pest of maize around the world; in particular *Sesamia nonagrioides* Lefèbvre, named Mediterranean corn borer (MCB), causes important losses in Southern Europe. Methods of selection can be combined with transgenic approaches to increase the efficiency and durability of the resistance to corn borers. Previous studies of the genetic factors involved in resistance to MCB have been carried out using bi-parental populations that have low resolution or using association inbred panels that have a low power to detect rare alleles. We developed a Multi-parent Advanced Generation InterCrosses (MAGIC) population to map with high resolution the genetic determinants of resistance to MCB.

**Results:**

We detected multiple single nucleotide polymorphisms (SNPs) of low effect associated with resistance to stalk tunneling by MCB. We dissected a wide region related to stalk tunneling in multiple studies into three smaller regions (at ~ 150, ~ 155, and ~ 165 Mb in chromosome 6) that closely overlap with regions associated with cell wall composition. We also detected regions associated with kernel resistance and agronomic traits, although the co-localization of significant regions between traits was very low. This indicates that it is possible the concurrent improvement of resistance and agronomic traits.

**Conclusions:**

We developed a mapping population which allowed a finer dissection of the genetics of maize resistance to corn borers and a solid nomination of candidate genes based on functional information. The population, given its large variability, was also adequate to map multiple traits and study the relationship between them.

## Background

The yield of crops is limited by several biotic factors such as weeds, animal pests, and pathogens. In maize, the average loss due to animal pests is 10% worldwide, according to data collected from 2001 to 2003, although there is large variation between regions [1]. A set of lepidopterans species grouped as corn borers by its effect on plants are animal pests with a high potential to reduce grain yield in maize. Thus, yield losses due to *Ostrinia nubilalis* (Hübner), an important corn borer found in Europe and in America and known as European corn borer (ECB), can reach, without control measures, 30% of the total maize production [2]. In the European Mediterranean area, *Sesamia nonagrioides* Lefèbvre*,* known as Mediterranean corn borer (MCB), coexists with ECB and is capable of doing a higher damage to the plants [3]. Climate change is expected to increase the frequency and intensity of biotic stresses [4]; more specifically, MCB is expected to spread out of its current area [2] as warmer climatic conditions probably favor this pest of African origin [5].

Early generations of corn borers damage the leaves, generally at early plant stages, while generations of corn borers damage the stalks once the plant has completed its vegetative development. The damage caused to stalks is usually more critical for yield than the damage produced in younger leaves [3]. Although MCB prefers attacking stalks, it can also produce direct kernel injury [6], damaging up to 48% of the ears [7].

Several defense mechanisms against insect herbivory have been found in maize [8] that can be broadly grouped into structural and biochemical defenses [9]. Structural mechanisms confer protection against insect damage by mechanical constrain to feeding or by nutrients dilution [10]. Resistance to corn borers has been related to cell wall composition and structure, particularly lignin content and composition and cross-linking of lignin to structural polysaccharides [11–14].

There could be trade-offs between plant growth and defense against insect herbivory [15], which in the case of crops means that the improvement of the plant defenses could be at the cost of yield and vice-versa. Specifically, for corn borer resistance it has been found that selection for resistance can be detrimental for grain yield and vice-versa [16, 17]. However, in other studies the correlation between resistance and yield was low or nonexistent [18–20] suggesting that the relationship between the two traits could be dependent on the specific properties of the populations. Flowering and plant height could be also correlated with stalk tunneling, although the degree of the relationship, from null to moderate, depended also on the population under study [18–20].

Transgenic or genetically modified crops that produce insecticidal compounds are a very efficient way to control the damage produced by insect pests, particularly corn borers, although the benefits are reduced by evolution of insect resistance [21]. These authors propose the combination of transgenic with other control tactics in integrated pest management to increase the durability of transgenic resistance. Recently, it was reported the first detection of a MCB resistance allele to *Bt* in Europe [22]. In addition, the cultivation of transgenic crops in some regions, for example Europe, is limited due to the suspicions raised by the technique [23]. Within integrated pest management, selection methods based on phenotypic evaluation can increase the resistance [24–26]. However, those selection methods are time and labor consuming because artificial infestation is required to guarantee a uniform attack in all genotypes [27]. Selection methods based on genomic information are effective in increasing resistance and can reduce the labor required for larva management and data collection [28–30].

Maize resistance to stalk tunneling by corn bores is polygenic probably due to the multiple mechanisms that are involved in resistance [31, 32]. The identification of the genes controlling the resistance would allow the improvement of the resistance by direct modification of the DNA sequences of individual genes by genome editing technologies as CRISPR/Cas [33]. However, although many QTL experiments have been carried out to map the genetic determinants of corn borer resistance, the individual genes behind the QTL have not been cloned so far. In several experiments, the mapping population was derived from two parents diverging for stalk tunneling, but the number of QTLs detected was relatively low and the QTLs were usually not consistent across populations. Besides, the confidence interval for the location of QTLs was large and many genes are included in those regions, which precludes reasonable proposals of the causal genes of the QTL. In some of the bi-parental populations, kernel damage was also analyzed, but a low number of QTLs were detected probably due to the low variability for the trait because the parents were selected for stalk tunneling. Association mapping of QTLs for resistance to MCB was carried out in a sample of inbred lines not selected for resistance [34] in order to overcome some of the limitations of the bi-parental experiments. In general, in the studies of association mapping, the confidence intervals of the QTLs are short due to the historical recombination, multiple alleles are analyzed, and QTLs can be detected for multiple traits [35]. However, the association studies have limited power to analyze rare alleles, which can be the most interesting for breeders [36–38]. Furthermore, in the statistical analysis of association mapping, a correction for population structure has to be made to avoid spurious signals, but the analysis may not always be able to completely avoid those false positives [39].

An advantage of mapping with Multi-parent Advanced Generation InterCrosses (MAGIC) populations over mapping with bi-parental populations is the ability to analyze several alleles simultaneously. The main advantages of MAGIC populations over association panels are the lack of an underlying unknown structure and the sufficient replication of all alleles to allow the statistical estimation of their effects [40, 41]. MAGIC populations can be integrated in breeding schemes, which make them also interesting for the private sector [42]. The selection of the parents to build the MAGIC population is critical and depends on the objectives of the research [43]. The integration of QTL detection and breeding was a priority for MAGIC populations developed in rice [44] and wheat [45, 46]. However, the research of biological processes was optimized in a MAGIC population developed in maize from parents of diverse origin and different heterotic groups [47].

We developed a MAGIC population with eight temperate maize inbred lines of diverse genetic origin, as five of them derive directly from different open-pollinated varieties from Spain, Italy, and France, while two lines are from Northern North America. All the parental lines belong to the No Stiff Stalk heterotic group and new inbreeds developed from them could have practical interest as they are expected to maintain high heterosis with the Stiff Stalk heterotic group. The parental lines were selected because they were the most resistant to MCB stalk tunneling in a previous evaluation of 121 inbred lines of temperate maize [31]. The objective of the present work is to map with high resolution genetic determinants of maize defense mechanisms against MCB attack using a MAGIC population. In addition, important agronomic traits such as days to silking, plant height, and yield were analyzed, to allow us to elucidate the relationship between those traits and resistance to MCB attack.

## Results

### The MAGIC population and its parents

**Fig. 1:**
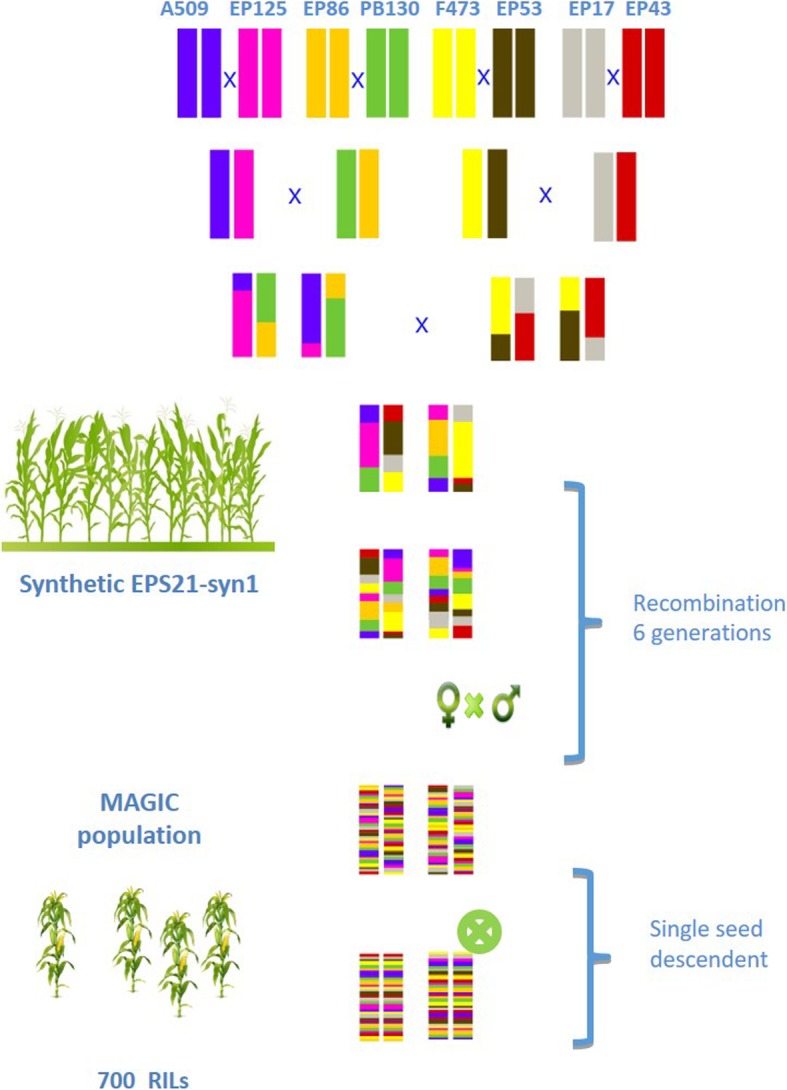
Scheme for the development of the MAGIC population

**Fig. 2:**
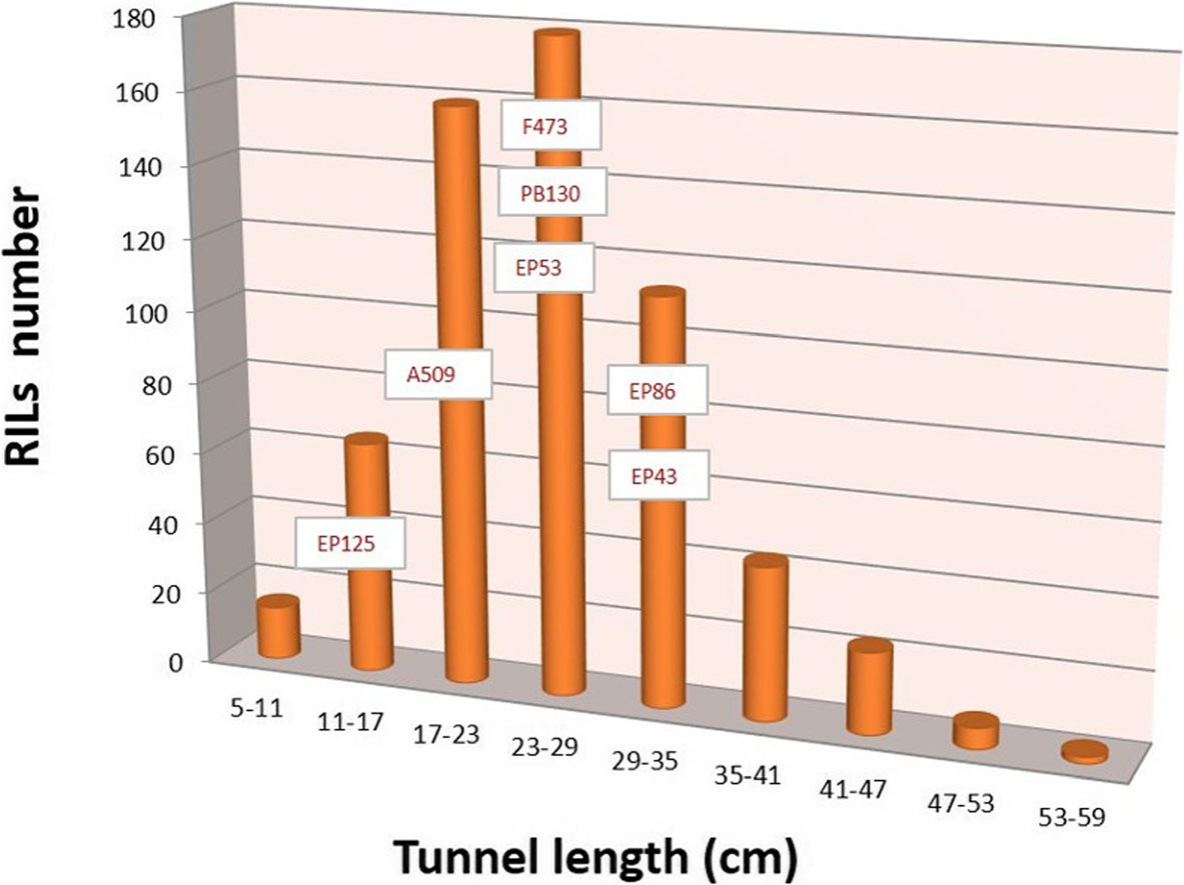
Distribution of stalk tunneling values in the RILs of the MAGIC population

The stalk tunnels in the plants of the susceptible control for stalk tunneling (EP42) spanned across one third of the total size of the plants, reaching an average value of 50 cm per plant that was significantly longer than the tunnels in the parents of the MAGIC population (Table 1). The length of the stalk tunnels varied among the parent of the MAGIC population which could be roughly grouped into three groups according to a size of tunnels of approximately 10 (EP125), 20 (EP53, A509, PB130, and F473), and 30 (EP86 and EP43) cm. For stalk tunneling, the RILs from the MAGIC population had a wide range of values, which exceeded the parents at the two extremes of the distribution (Fig. 1). Regarding kernel resistance, all parents had a value of the visual scale higher that the average (5). PB130 had the lowest value of kernel resistance (6.1) among the parents, although the values of F473 and EP43 (6.5–6.7) did not differ statistically from the value of PB130. On the other hand, the kernel resistance of the remaining parents and EP42 was significantly higher than PB130 (7.2–7.7). The kernel resistance of many of the RILs concentrated around the values of the parents (6–8), although a considerable number of RILs exceeded the value of the parents (8–9) (Fig. 2). On the contrary, the number of RILs with values of kernel resistance lower than that of the parents was low, especially when the values moved away from the values of the parents. Thus, the number of RILs with a kernel resistance lower than 5 was extremely reduced. For days to flowering and for plant height, the values of the RILs of the MAGIC population were placed mainly between the values of the parents, which had wide variability for these traits (Additional file 1: Figure S1b-c). For grain yield, all the parents, except EP43, had values between 33 and 43 g plant^− 1^ that were not statistically different. The RILs of the MAGIC population had large variation for grain yield and a considerable number of them exceeded the parents (Additional file 1: Figure S1a). The heritability was high for plant height and days to flowering, moderate for yield, and low for traits related to resistance. The correlation between defense and agronomic traits was low (data not shown).

**Table 1:** Means ± standard errors and heritabilities (*h*^2^) of the MAGIC population for agronomic and MCB resistance traits. The means of the eight parents of the MAGIC population and a control (EP42) which is susceptible to stalk tunneling are also shown

	Resistance traits		Agronomic traits		
	TL (cm)	KR (1–9)^a^	S (days)	Y (g plant^−1^)	PH (cm)
RIL					
Mean	24.6 ± 0.36	7.4 ± 0.03	69.8 ± 0.15	40.0 ± 0.46	147.6 ± 0.73
*h*^*2*^	0.30	0.24	0.84	0.53	0.82
Parents and controls					
EP42	50.1 a	7.3 abc	67.1 cde	42.4 a	159.7 ab
EP17	–	–	–	–	–
EP125	12.8 d	7.7 a	64.8 ef	42.6 a	141.0 bcd
EP86	32.9 b	7.2 abc	70.4 abc	40.3 a	181.0 a
EP53	24.9 c	7.5 ab	62.5 f	39.9 a	132.7 cd
A509	22.3 c	7.4 abc	65.9 def	34.0 a	126.3 d
PB130	24.2 c	6.1 d	69.5 bcd	33.5 a	154.8 bc
F473	23.2 c	6.7 bcd	74.2 a	33.4 a	150.1 bcd
EP43	30.2 bc	6.5 cd	73.2 ab	11.0 b	161.5 ab
LSD *P* > 0.05	7.9	0.9	4.0	12.4	25.5

*TL* Tunnel length, *KR* Kernel resistance, *S* Days to silking, *Y* Grain yield, *PH* Plant height. The heritabilities (*h*^*2*^) for each trait were estimated following [48]

^a^The kernel resistance was measured with a subjective visual scale of 1 to 9 in which 1 indicates completely damaged and 9 indicates no damage

**Fig. 3:**
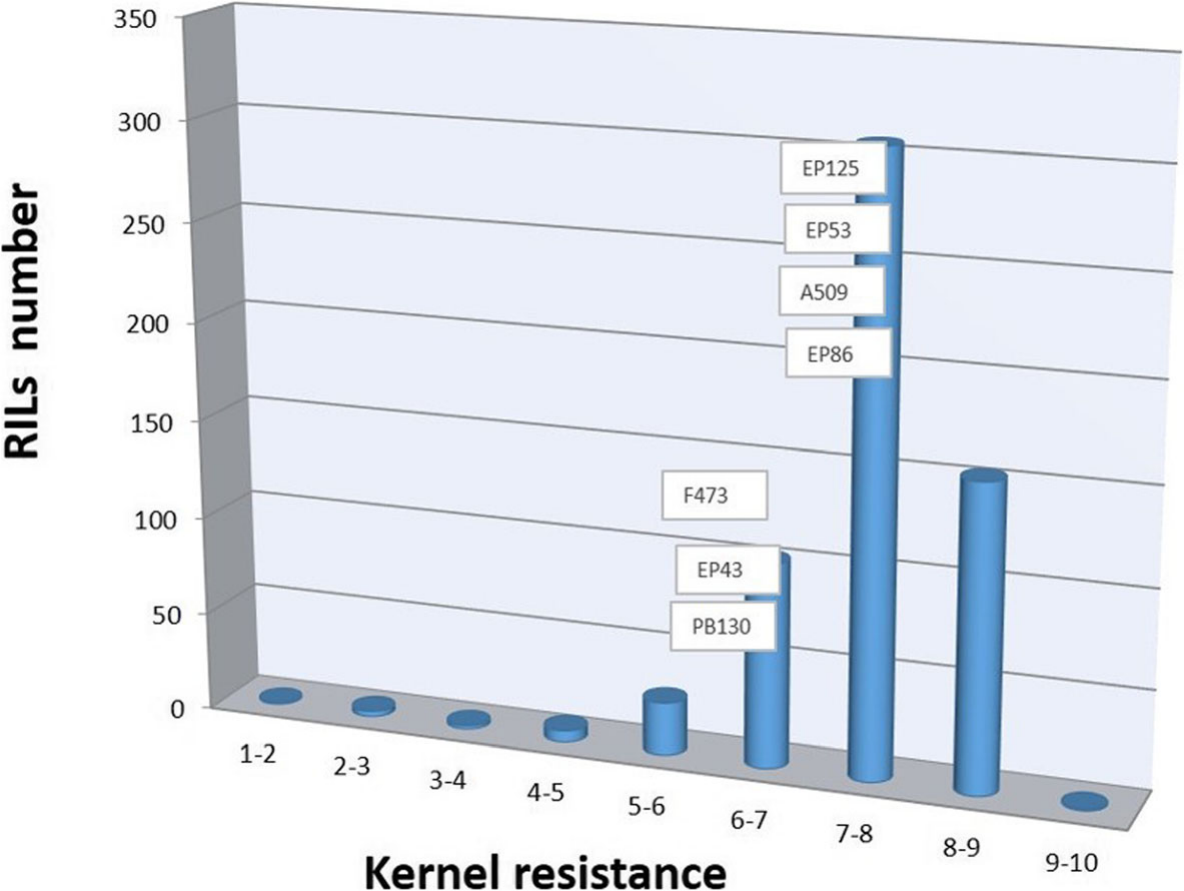
Distribution of kernel resistance values (scale from 1=complete damage to 9=no damage) in the RILs of the MAGIC population

For the RILs and parents of the MAGIC population, the terminal branches of the Neighbor joining cladogram were long with the common ancestors placed near the center of the cladogram (Additional file 2: Figure S2). In the principal component analysis, the proportion of variance explained by the two principal axes was low and no evidence of subgroups is observed in the graph (Fig. 3). Regarding the linkage disequilibrium of the population, the length of the haplotype blocks was lower than 0.5 Mb; R^2^ decayed rapidly reaching values equal to or lower than 0.1 after 1.0–1.5 Mb (data not shown).

### Genomic regions associated with stalk tunneling

Twenty-seven SNPs distributed along all chromosomes, except chromosomes 9 and 10, were significantly associated with stalk tunneling (Table 2). Among those, S3_218807815, S4_155830370, S4_155830400, S8_28526020 and S8_28526026 were close (within 3000 bp) to other SNPs associated with the trait. We will focus on the 22 remaining significant SNPs for further analysis and discussion. The additive effects for individual SNPs ranged between 1.5 and 2.5 cm in most of the SNPs. Only three SNPs had an additive value higher than 2.5 cm, particularly S4_221752511 that had the highest value (3.7 cm). The percentage of variance explained by individual SNPs was 3% for 12 of the SNPs, between 4 and 5% for 9 of the SNPs, and 6% for 1 SNP.

**Table 2:** SNPs significantly associated with resistance to stalk tunneling by MCB. The genotype of the parents, the number of RILs with favorable and unfavorable alleles, the additive value, the significance of the association, and the variance explained by each SNP are included in the table. Previous experiments with QTL detected in the same bin that the significant SNPs are shown in the last column of the table

Significant SNP^a^	bin	A509	EP125	EP17	EP43	EP53	EP86	F473	PB130	Alleles^b^	(No)^c^	Additive effect^d^	*p-value* ^e^	R^2 f^	Previous experiments with co-localizing QTL^g^
S1_19252698	1.02	C	T	T	C	C	C	C	T	C/T	381/132	1.65	4.6E-05	0.03	MCB EP42xEP39 [17]; MCB EP125xPB130 [49]
S1_290934634	1.11	T	T	T	C	T	N	T	N	C/T	64/274	2.13	9.9E-05	0.05	
S2_14798875	2.02	T	C	N	C	C	C	N	C	T/C	212/242	1.48	9.76E-05	0.03	
S2_179803199	2.07	A	G	G	G	G	N	G	A	A/G	170/348	1.49	9.9E-05	0.03	ECB B73xB52 [50]; ECB B73xDe811 [51]; MCB EP125xPB130 [49]
S3_191332395	3.07	N	G	N	N	G	C	N	G	C/G	138/269	2.01	4.7E-06	0.05	ECB European Dent [30]
S3_212770896	3.08									G/C	60/451	2.16	7.16E-05	0.03	
S3_218807815	3.09	G	G	G	G	G	G	N	T	G/T	396/81	1.87	9.4E-05	0.03	ECB D06xD408 [52]
S3_218807820	3.09	G	G	G	G	G	G	N	A	G/A	397/80	1.92	6.5E-05	0.03	
S4_127856740	4.05	T	N	G	G	N	G	G	G	G/T	372/97	1.88	8.0E-05	0.03	
S4_127955231	4.05	A	A	G	G	N	G	G	G	G/A	399/128	1.77	3.6E-05	0.03	
S4_155128691	4.06	T	T	T	N	T	N	T	T	G/T	63/345	2.38	1.2E-05	0.05	
S4_155830369	4.06	C	N	C	N	C	C	N	C	T/C	43/361	2.55	3.2E-05	0.05	
S4_155830370	4.06	T	N	T	N	T	T	N	T	G/T	43/361	2.55	3.2E-05	0.05	
S4_155830400	4.06	G	N	G	N	G	G	N	G	T/G	43/361	2.55	3.2 E05	0.05	
S4_156193095	4.06	C	C	C	N	N	C	T	C	T/C	78/308	2.32	2.8E-06	0.06	
S4_181340312	4.09	C	T	N	N	C	T	N	N	T/C	207/144	1.69	9.3 E05	0.04	
S4_221752511	4.09	N	N	G	G	N	G	G	G	A/G	19/282	3.68	7.1 E05	0.05	
S5_24771445	5.03	A	N	G	G	G	A	G	G	G/A	345/117	1.82	2.4E-05	0.04	ECB D06xD408 [52]; MCB B73xCML103 [20]
S6_147725553	6.05	A	A	N	A	N	N	N	N	A/T	401/49	2.50	4.6E-05	0.04	ECB B73xDe811 [51]; MCB B73xCML103 [20]; MCB EP125xPB130 [49]
S6_150800759	6.05	A	A	N	A	G	G	G	G	A/G	247/264	1.42	7.0E-05	0.03	
S6_156035854	6.06	A	N	A	A	A	N	C	C	A/C	356/179	1.46	8.2E-05	0.03	ECB D06xD408 [52]; MCB EP125xPB130 [49]
S6_164776991	6.07	N	A	G	A	G	G	A	G	G/A	347/168	1.55	4.5E-05	0.03	ECB B73xMo47 [50]; MCB EP125xPB130 [49]
S7_109722251	7.02	G	A	A	A	N	N	G	N	G/A	244/246	1.42	9.9E-05	0.03	ECB B73xB52 [53]
S8_24527783	8.03	N	G	G	G	N	N	G	G	T/G	27/393	3.00	9.4E-05	0.04	ECB B73xMo47 [50]
S8_28525990	8.03	T	G	G	G	G	G	G	G	T/G	59/502	2.16	8.2 E05	0.03	
S8_28526020	8.03	A	C	C	C	C	C	C	C	A/C	59/503	2.16	8.1E-05	0.03	
S8_28526026	8.03	C	A	A	A	A	A	A	A	C/A	59/503	2.16	8.1 E05	0.03	

^a^The number before the underscore (_) indicates the chromosome number and the number after the underscore (_) indicates the physical position of the SNP in bp within the chromosome

^b^The allele before the slash (/) increases the trait and the allele after the slash decreases the trait

^c^No = number of homozygous lines for a given variant. The number before the slash refers to the allele that increases the trait and the number after the slash to the allele that decreases the trait

^d^The additive effect was calculated as half the difference between the mean of the homozygotes for the allele that increases the trait and the mean of the homozygote that decreases the trait

^e^The significance threshold based on the deviation of F observed from expected is p = 1 × 10^−4^

^f^R^2^, proportion of the phenotypic variance explained by the SNP

^g^In the first place we show the corn borer species, in the second place the mapping population, and in the third place the reference. Different experiments are separated by semicolons

The number of favorable alleles was higher than the number of unfavorable alleles for all parents except A509 and EP43. EP125, that had the shortest tunnels, showed the highest number of favorable alleles (13), while EP43 and EP86, that had the longest tunnels, showed the lowest number of favorable alleles (7–8). A509, F473, EP17, and EP53, that had tunnels of intermediate size, had also an intermediate number of favorable alleles (9–10). However, PB130 had tunnels of intermediate size, but the highest number of favorable alleles (together with EP125). The total number of favorable and unfavorable alleles detected in all parents was 79 and 52, respectively.

When the genotype of any of the parents was missing, the frequency of favorable alleles in the parents was inferred from the frequencies in the MAGIC population assuming that the selection and random drift did not change the allele frequency during the development of the MAGIC population. We considered that in the parental lines, for each SNP, there was one favorable allele and one unfavorable allele repeated in different proportions, for example 1 favorable vs 7 unfavorable alleles. In two of the significant SNPs associated with stalk tunneling, only one of the parents had the favorable allele; for the remaining significant SNPs the favorable allele was shared for more than one parent. In six of the SNPs with favorable alleles shared by the parents, all parents but one had favorable alleles.

### Genomic regions associated with kernel resistance

Twenty-three SNPs distributed in all chromosomes, except chromosome 8, were significantly associated with kernel resistance (Table 3). Five significant SNPs associated with kernel resistance were very close (within 3000 bp) to SNPs also significantly associated with the trait and were not considered in further analyses and discussion. Only one SNP significantly associated with kernel resistance, S6_165637552, was close (within 1 ~ Mb) to a SNP significantly associated with stalk tunneling. The additive value associated with individual SNPs ranged between 0.15 and 0.46. Most of the SNPs explained between 3 and 5% of the variance, but the variance explained by S3_220658669 reached 9%.

**Table 3:** SNPs significantly associated with kernel resistance to MCB attack. The genotype of the parents, the number of RILs with favorable and unfavorable alleles, the additive value, the significance of the association, and the variance explained by each SNP are included in the table. Previous experiments with QTL detected in the same bin that the significant SNPs are shown in the last column of the table

Significant SNP^a^	bin	A509	EP125	EP17	EP43	EP53	EP86	F473	PB130	Alleles^b^	(No)^c^	Additive effect^d^	*p-value* ^*e*^	R^2 f^	Previous experiments with co-localizing QTL^g^
S1_11476253	1.01	N	N	N	G	A	G	A	A	A/G	282/88	0.22	9.3E-05	0.04	
S1_29155773	1.02	C	C	N	N	N	C	T	C	C/T	323/37	0.32	4.9E-05	0.04	
S1_37531738	1.03	C	C	C	G	N	N	N	C	C/G	367/23	0.39	5.7E-05	0.04	
S1_295188904	1.12	G	G	G	G	G	N	N	N	G/A	322/32	0.35	3.4E-05	0.05	MCB EP42xEP39 [18]
S2_20109642	2.01	G	G	N	G	N	N	G	G	G/C	389/28	0.42	2.2E-06	0.05	
S2_202280633	2.07	G	N	G	G	N	N	N	G	G/A	350/21	0.41	4.9E-05	0.05	
S2_213160826	2.08	N	A	A	A	A	N	A	A	A/T	447/33	0.33	5.3E-05	0.03	
S3_220658669	3.09	G	G	G	G	N	N	G	G	G/A	278/28	0.46	6.7E-07	0.09	MCB Association panel [34]
S3_220658703	3.09	A	A	A	A	N	A	A	A	A/G	326/20	0.47	7.2E-06	0.06	
S4_236927609	4.09	T	T	N	T	N	N	A	T	T/A	173/136	0.21	3.9E-05	0.06	MCB Association panel [34]
S5_1442632	5.00	G	G	G	A	G	A	G	N	G/A	391/104	0.21	3.3E-05	0.04	
S5_1848216	5.00	C	C	C	C	N	N	C	N	C/T	396/29	0.34	9.3E-05	0.04	
S5_9090576	5.01	G	G	G	T	T	G	G	G	G/T	445/132	0.19	3.3E-05	0.03	MCB B73xCML103 [20]
S6_165637552	6.07	C	N	C	N	C	C	C	C	C/A	448/26	0.36	9.4E-05	0.03	
S7_955804	7.00	C	C	C	C	C	C	C	N	C/A	428/34	0.32	3.7E-05	0.04	
S7_958414	7.00	N	C	N	C	N	C	C	N	C/T	448/38	0.32	2.2E-05	0.04	
S7_958454	7.00	N	C	N	C	C	C	C	N	C/T	488/45	0.27	8.8E-05	0.03	
S7_1952227	7.00	T	T	N	T	N	T	N	C	T/C	312/113	0.22	9.5E-06	0.05	
S7_1952249	7.00	G	G	N	G	N	G	N	A	G/A	312/113	0.22	9.5E-06	0.05	
S9_37401486	9.01	G	N	N	N	G	G	N	G	G/T	325/58	0.26	4.7E-05	0.04	
S9_37401504	9.01	C	N	N	N	C	C	N	C	C/G	325/58	0.26	4.7E-05	0.04	
S9_147511039	9.06	G	N	N	G	G	N	G	N	G/A	353/24	0.41	1.5E-05	0.05	MCB A637xA509 [54]
S10_136106669	10.05	A	N	N	A	G	N	A	N	A/G	350/28	0.39	1.3E-05	0.06	

^a^The number before the underscore (_) indicates the number of the chromosome and the number after the underscore (_) indicates the physical position of the SNP in bp within the chromosome

^b^The allele before the slash (/) increases the trait and the allele after the slash decreases the trait

^c^No = number of homozygous lines for a given variant. The number before the slash is for the allele that increases the trait and the number after the slash for the allele that decreases the trait

^d^The additive effect was calculated as half the difference between the mean of the homozygotes for the allele that increases the trait and the mean of the homozygote that decreases the trait

^e^The significance threshold based on the deviation of F observed from expected is p = 1 × 10^− 4^

^f^R^2^, proportion of the phenotypic variance explained by the SNP

^g^In the first place we show the corn borer species, in the second place the mapping population, and in the third place the reference. Different experiments are separated by semicolons

All parents had higher number of favorable alleles than unfavorable alleles for kernel resistance. EP125 and A509 had a higher number of favorable alleles, a lower number of unfavorable alleles, and more kernel resistance than EP43, F473, and PB130. However, EP86 and EP53 had the lowest number of favorable alleles, but their kernel resistance was similar to that of EP125 and A509. The total number of favorable alleles detected in all parents was 85, while the number of unfavorable alleles was 11. For most of the SNPs, six or seven of the parents shared the favorable allele. For several of the SNPs associated with kernel resistance, the frequency of the less common allele was lower (0.06–0.07) than the value expected if only one parent had the allele (0.125).

### Genomic regions associated with agronomic traits

**Fig. 4:**
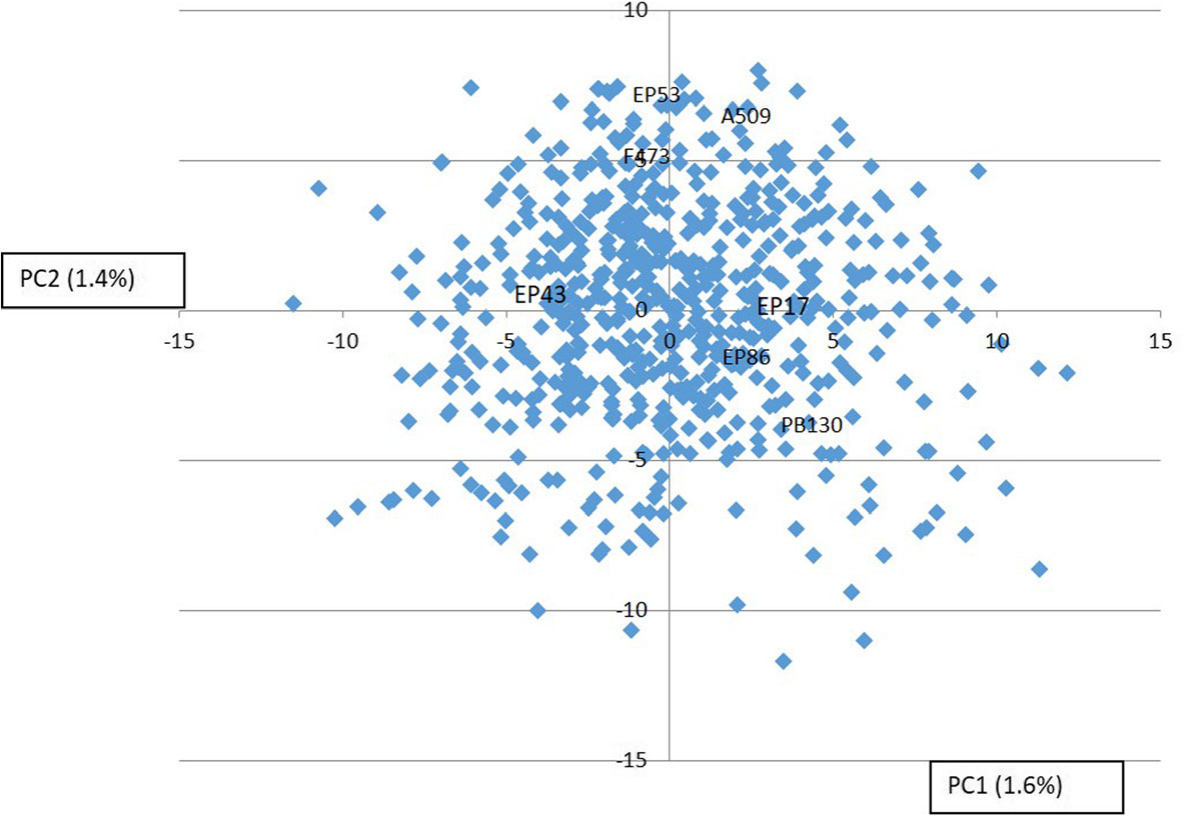
Principal component analysis of the SNPs from the RILs of the MAGIC population

**Fig. 5:**
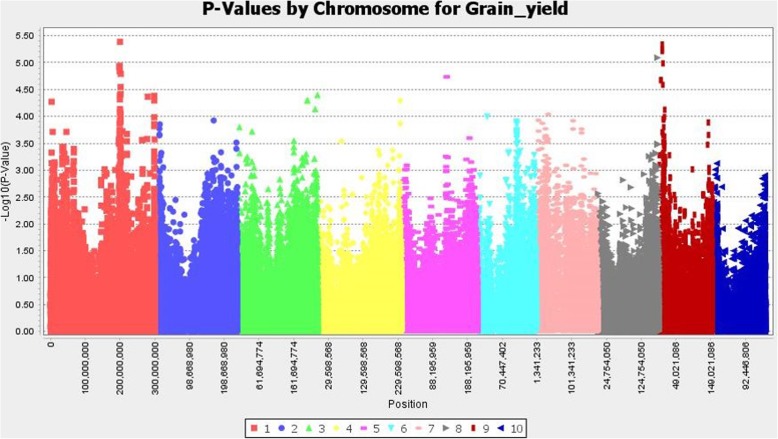
Association between SNPs and grain yield. The significance threshold based on deviation of F observed from expected (p = 10^− 4^) is shown as a horizontal line

**Fig. 6:**
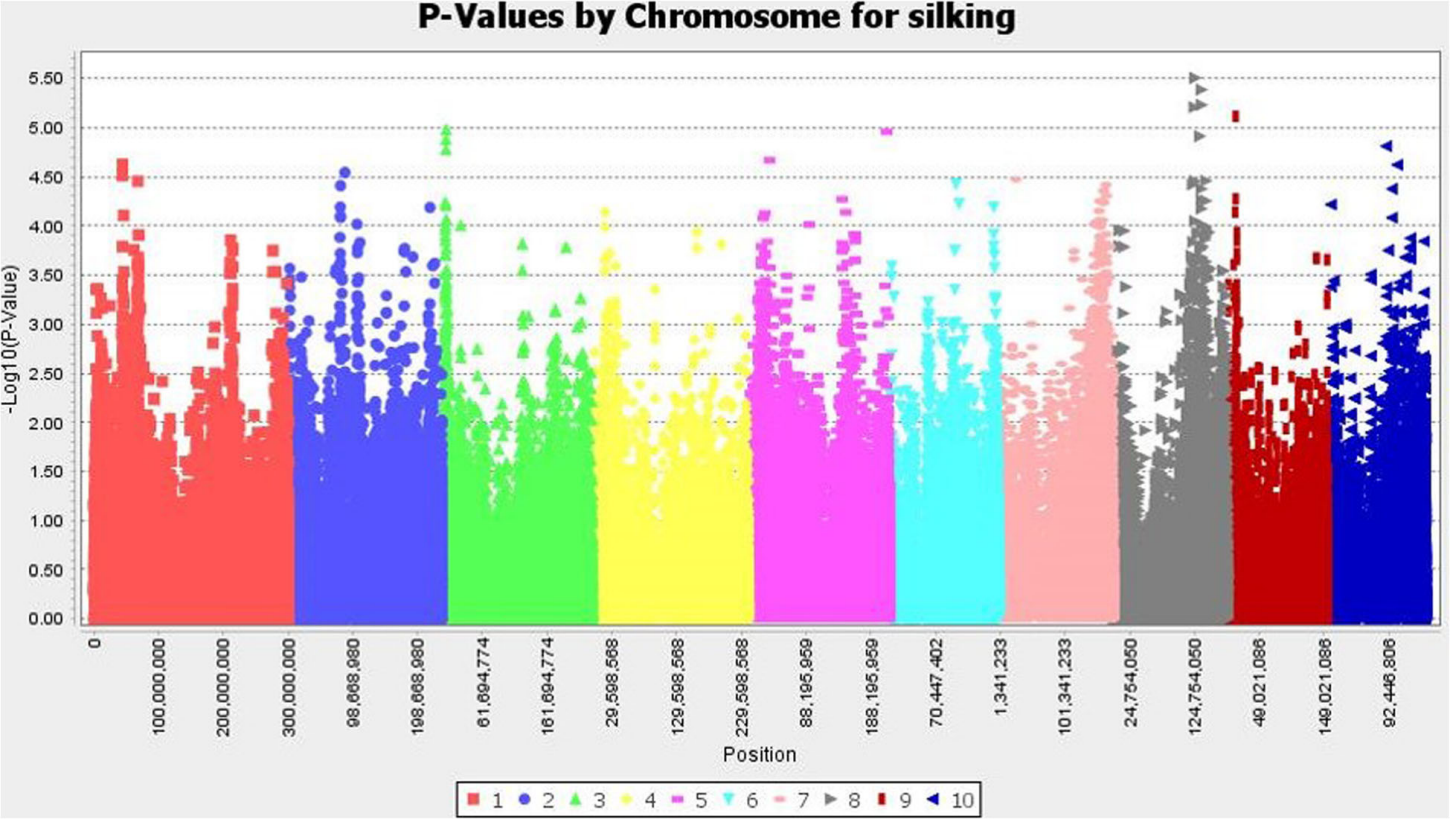
Association between SNPs and days to flowering. The significance threshold based on deviation of F observed from expected (p = 10^− 4^) is shown as a horizontal line

We detected several significant SNPs associated with grain yield under high pressure of corn borer, days to flowering, and plant height (Figs. 4, 5, 6, and Additional file 3: Table S1a-c). The percentage of variance explained by individual SNPs ranged between 3 and 6%. We did not found significant SNPs associated with agronomic traits co-localized with significant SNPs associated with resistance traits, with the exception of one SNP in common for stalk tunneling and flowering time.

## Discussion

### The MAGIC population and its parents

The parents of the MAGIC population, as expected given their diverse genetic origin, had wide variability for several traits. The RILs from the MAGIC population had also a wide range of values for the different traits that exceeded the parents in the two tails of the distribution. Although all parents were resistant to stalk tunneling, there was transgressive segregation in the MAGIC population and some RILs were susceptible; which indicates that the parents should carry both favorable and unfavorable alleles for resistance. The heritabilities found in this experiment are consistent with previous studies, that showed that the agronomic traits, particularly days to flowering and plant height, have higher heritabilities than traits related to resistance to corn borers [20, 34, 54].

In the development of 8-way MAGIC populations, the inbred founders are paired off and inter-mated in a prescribed order for each line, known as funnel [43]. Different funnels are usually combined and as a result of this, different 8-way families are generated which are self-pollinated without further recombination. We used a different scheme consisting of a single funnel without replication that resulted in a single type of an 8-way family that was recombined at random during 6 cycles before self-pollination. Although a limited number of funnels in our MAGIC population could have created a structure in the population and biased the analysis [43], neither the neighbor joining cladogram nor the principal component analysis showed signs of structure in the MAGIC population. The 6 cycles of random recombination probably contributed to the homogenization of the population.

In our MAGIC population, higher number of associations between markers and traits were detected than in bi-parental populations, particularly for traits not used to select the parents of the mapping population. Pascual et al. [55] also found more associations in a MAGIC population than in a bi-parental population in tomato. Meng et al. [44] found in rice that two 4-way MAGIC populations failed to detect associations detected in an 8-way MAGIC population that covers the eight parents of the 4-way populations. According to the authors, the advantage of the 8-way mapping population to detect associations between markers and traits was due to the higher number of comparisons between different alleles. For sizes similar to our MAGIC population, simulations of MAGIC populations in maize and Drosophila found moderate to high power of detection for QTL of minor effect [47, 56]. In the simulations by Klasen et al. [57] a reduction in power was observed when a large number of QTLs controlled the character, although the mapping populations with higher number of parental lines tended to have a higher power of QTL detection. Our MAGIC population had also higher resolution than previous bi-parental populations due to the recombination cycles and the large population size.

### Genomic regions associated with stalk tunneling

The effects of the individual SNPs for stalk tunneling were lower than in previous studies [18, 20, 54, 58]. The size of the bi-parental mapping populations used in previous studies was relatively low (200 individuals, approximately), which leads to inflated estimates of QTL effects [59]. The higher size of our MAGIC population allows a more precise estimation of effects that turned out to be very low for many of the associations (R^2^ = 0.03). Such genetic architecture suggests, in agreement with [30], that genomic selection would be more efficient than selection methods based exclusively on markers linked to significant QTLs. All lines had favorable alleles for resistance to stalk tunneling as expected given that all of them have some level of resistance. For most of the SNPs, the favorable alleles were shared by some of the parents indicating that the parents, in spite of their diverse origins, share common resistance mechanisms. Bandillo et al. [60] selected each of the parents of a MAGIC population as a donor of a major trait and found specific sources of resistance or tolerance in the parents not selected as donors for that type of resistance.

Four genomic regions (1.02, 2.06, 5.03 and 6.05/6.07) associated with stalk tunneling in our experiment were also repeatedly identified in other experiments. Santiago et al. [49] detected only three regions associated with stalk tunneling that were close to regions detected in our experiments (1.02, 2.06 and 6.05/6.07). This was expected because the mapping population used by those authors was developed from two parents of the MAGIC population. However, QTLs for stalk tunneling were also found in those regions or in their proximity in other experiments using unrelated mapping populations (Table 2). In addition, it was found that three of the four regions (1.02, 5.03, 6.05/6.07) co-localized to QTLs are related to cell wall digestibility [61]. The wide region covering from 6.05 to 6.07 is an important region associated with cell wall components in multiple experiments [51, 62, 63]. Fine studies with multiple bi-parental populations and association panels have disentangled the region to at least three QTLs: one at ~ 165 Mb, other at ~ 150 Mb, and finally other between them, probably at ~ 155 Mb [64–67]. The region 6.05–6.07 has been also associated with resistance to stalk tunneling in different mapping populations and environments. With the MAGIC population, we dissected the region into three small sub-regions around the significant markers S6_150800759, S6_156035854, and S6_164776991, that closely overlap (within ~ 1 Mb) with the three regions associated with cell wall digestibility at ~ 150, ~ 155, and ~ 165 Mb. Candidate genes proposed for the three cell wall QTLs could be the causal genes for the stalk tunneling QTLs that co-localized with them in our experiment. Thus, a candidate gene for the QTL at ~ 165 Mb could be GRMZM2G031200, which is located ~ 0.3 Mb away from the significant SNP and is a NAC transcription factor involved in secondary cell wall biosynthesis [68]. One possible candidate gene for the QTL at 150 is the laccase gene GRMZM2G146152, orthologous to AtLac17 that affects lignin content [65] and is located ~ 0.7 Mb away from the significant SNP. Regarding the genes in the region of the QTL at ~ 155 Mb, GRMZM2G140817 (C3H2) is located ~ 0.4 Mb distant from the significant SNP and is related to C3H1 that controls the phenylpropanoid flux. The down regulation of C3H1 increases the amounts of p-hydroxyphenyl (H) units of lignin [69] which, in turn, decreases the digestibility of the plant tissues [68].

The QTL for cell wall components at bin 1.02 was reduced to a smaller region (from ~ 12 to ~ 22 Mb) by joint analysis of different experiments [65]. A significant SNP associated with digestibility was also located within that region in the association analysis of [67], specifically at ~ 18 Mb. For this QTL, [65] proposed the candidate gene GRMZM2G109431 located at ~ 18 Mb which is homologous to AT4G3330 in Arabidopsis and related to secondary wall deposition. This gen is located ~ 1.5 Mb distant from S1_19252698, associated with stalk tunneling in our MAGIC population, and could be the gene responsible for the differences between alleles for stalk tunneling at that location.

The close co-localization of QTL related to stalk tunneling and relevant genes related to cell wall biosynthesis confirms the importance of the cell wall structure and composition in resistance to herbivores. This is consistent with previous phenotypic analysis of some of the parental lines of the MAGIC population because EP125 presented high concentration of simple hydroxycinnamates and diferulates, that could confer increased cell wall strength throughout cross-linking [12], and A509 showed thickened cell walls [70]. However, cell wall characteristics, although important, are not the only defense mechanisms against corn borers and alternative mechanisms could be behind some of the QTLs for stalk tunneling. For example, the most reasonable candidate gene for S3_191332395 QTL3_ST is GRMZM2G057140 located ~ 0.08 Mb away from the significant SNP. This gen is homologous to VIH2 of Arabidopsis (MaizeGDB) that regulates the synthesis of inositol pyrophosphate and jasmonate-depent defenses in this species [71]. These authors show that VIH2 mutants have decreased resistance against larvae of herbivorous insects.

### Genomic regions associated with kernel resistance

The distribution of favorable alleles was different for kernel resistance in comparison to stalk tunneling. For kernel resistance in most of the SNPs associated with the trait, the favorable allele was shared for seven of the parents, while for stalk tunneling in several of the SNPs the favorable allele was shared by a number of parents lower than seven. The choice of the parents based on their resistance to stalk tunneling has probably conditioned the type of alleles detected in other traits. Thus, for kernel resistance in most of the cases we are identifying deleterious alleles carried by specific parents. The presence of several SNPs associated with kernel resistance with lower than expected frequency of the less common allele suggests that the SNPs associated with kernel resistance went through unintentional natural selection during the development of the MAGIC population. A residual heterozygosity could also contribute to the low kernel damage showed by the RILs.

Only one SNP associated with kernel resistance, S6_165637552, was close (within ~ 1 Mb) to a SNP associated with stalk tunneling. This is consistent with the low correlation found between the two traits in our and previous experiments [3, 7, 31]. Our results confirm that, in general, different mechanisms are involved in kernel and stem resistance. S3_220658669 at 3.09 is the only SNP associated with kernel resistance with an effect relatively large, close to 10%, and was also detected in an association panel [34]. This significant SNP was embedded in a region where QTLs for kernel resistance to *Sitophilus zeamais* (Motsch.) (maize weevil) and diferulate content of kernel pericarp were previously located [72]. It seems that differences in the structure of the cell wall in the pericarp of the kernel are responsible for the differences in kernel resistance to maize weevil and probably to MCB as well. The hardness of the pericarp could hinder the feeding of the larvae from the kernels. The defense mechanism behind S6_165637552 could also be related to cell wall characteristics of stalk tissues instead of pericarp, as it co-localizes with a SNP related to stalk tunneling (S6_164776991), and the region 6.07 is an important region related to cell wall components and digestibility in stalk tissues, but not in pericarp kernels. The MCB larvae usually access the ear after feeding from the stem and the defense mechanism behind S6_164776991 probably reduces their access to the ears. The SNP S9_147511039 is located in the bin 9.07 where a QTL that explained a substantial part of the genetic variation (20%) for kernel resistance to MCB was detected in the A637 × A509 population [54]. S9_147511039 is located within the gene GRMZM2G178190, which is responsible for zinc metabolism in maize and could regulate its accumulation in grains [73]. The zinc content has been associated with larval survival and adult emergence in *Chilo partellus* (Swinhoe) [74]. Zinc is also a potent inhibitor of gut α-amylase in *Helicoverpa armigera* (Hübner) [75] causing the reduction in the energy reserves of the larvae [76]. Zinc also plays a role in maintaining the structural integrity and biological function of a cysteine protease inhibitor in *Pennisetum glaucum* (L.) R. Br. (pearl millet) that possesses anti-feeding activity [77].

### Genomic regions associated with agronomic traits

Three of the regions where significant SNPs associated with grain yield under high pressure of corn borer were detected (~ 199–202 Mb in chromosome 1, ~ 128 Mb in chromosome 5, and ~ 6–12 Mb in chromosome 9) were close (~ 0.2 Mb) or within 3 meta-QTL for grain yield detected by Wang et al. [78]. In chromosome 1, a meta-QTL containing a high number of individual QTLs and spanning between ~ 200 and ~ 208 Mb was also detected by Pan et al. [79]. In addition, the analysis of Wang et al. [78] identified a meta-QTL at ~ 197–198 Mb in chromosome 1, very close to the previously mentioned. In congruence with the presence of different QTLs located close to each other, we dissected the region of chromosome 1 in three smaller sub-regions at ~ 199, ~ 200, and ~ 202 Mb. The consistency of QTLs detected under high level of infestation and standard conditions suggests that those QTLs are not per se involved in resistance, but are related to grain yield. We should achieve an improved yield under optimal conditions, but also under conditions of high pest infestation by transmitting these QTLs through selection.

We found a large number of significant SNPs associated with days to flowering and plant height as expected given the high heritability of these traits. Flowering time is one of the traits that have been more deeply investigated at molecular level in maize and one of the few quantitative traits in which individual genes have been cloned so far. We detected a significant SNP associated with flowering at less than ~ 1 Mb from Znc8 (S8_124,357,599) and Zm-Rap2.7 (S8_132,878,255). Zcn8 and Zm-Rap2.7 were the candidate genes with more strong association to significant SNPs for flowering time in a study that characterized the diversity of 4471 maize landraces [80]. Zcn8 is orthologue of the Arabidopsis FT florigen that integrates endogenous and photoperiod flowering signals and is probably involved in the adaptation of maize to temperate climates [81–84]. The Vgt1 is a cis-regulatory element of the flowering gen Zm-Rap2.7 which was cloned by Salvi et al. [85] and it is probably also relevant for the adaptation of maize to temperate climates [86, 87].

The strength of the relationship between resistance and agronomic traits has implications on the simultaneous selection for both kinds of traits or on indirect selection of non-target traits when the selection is based on another trait. Also, the possibility of using markers associated with QTLs for stalk tunneling with breeding purposes depends on the side effects of the QTLs on agronomic traits. We found low correlation between defense and agronomic traits (data not shown) and did not found QTLs co-localizing for different traits, with the exception of one QTL in common for stalk tunneling and flowering time. Therefore, it is possible in our population to select for resistance against corn borers without a detrimental effect on agronomic traits.

## Conclusions

We developed a multi-parental mapping population of large size, which allowed a finer dissection of the genetics of maize resistance to corn borers, and a solid nomination of candidate genes based on functional information. The population, given its large variability, was also adequate to map agronomic traits and study its relationship with resistance traits. Our results indicate that multiple genetic factors are involved in the defensive response of the maize plant to corn borers. The favorable alleles for resistance were generally shared for some of the parents and thus, each resistant genotype is the result of the cumulated effect of several common favorable alleles. For some SNPs associated with resistance, genes related to the cell wall biosynthesis and assembly were strong candidates for explaining the association. When more information about the function of genes and its relationship with resistance be published, causal genes behind additional associations could be proposed.

## Methods

### Development of the MAGIC population

**Fig. 7:**
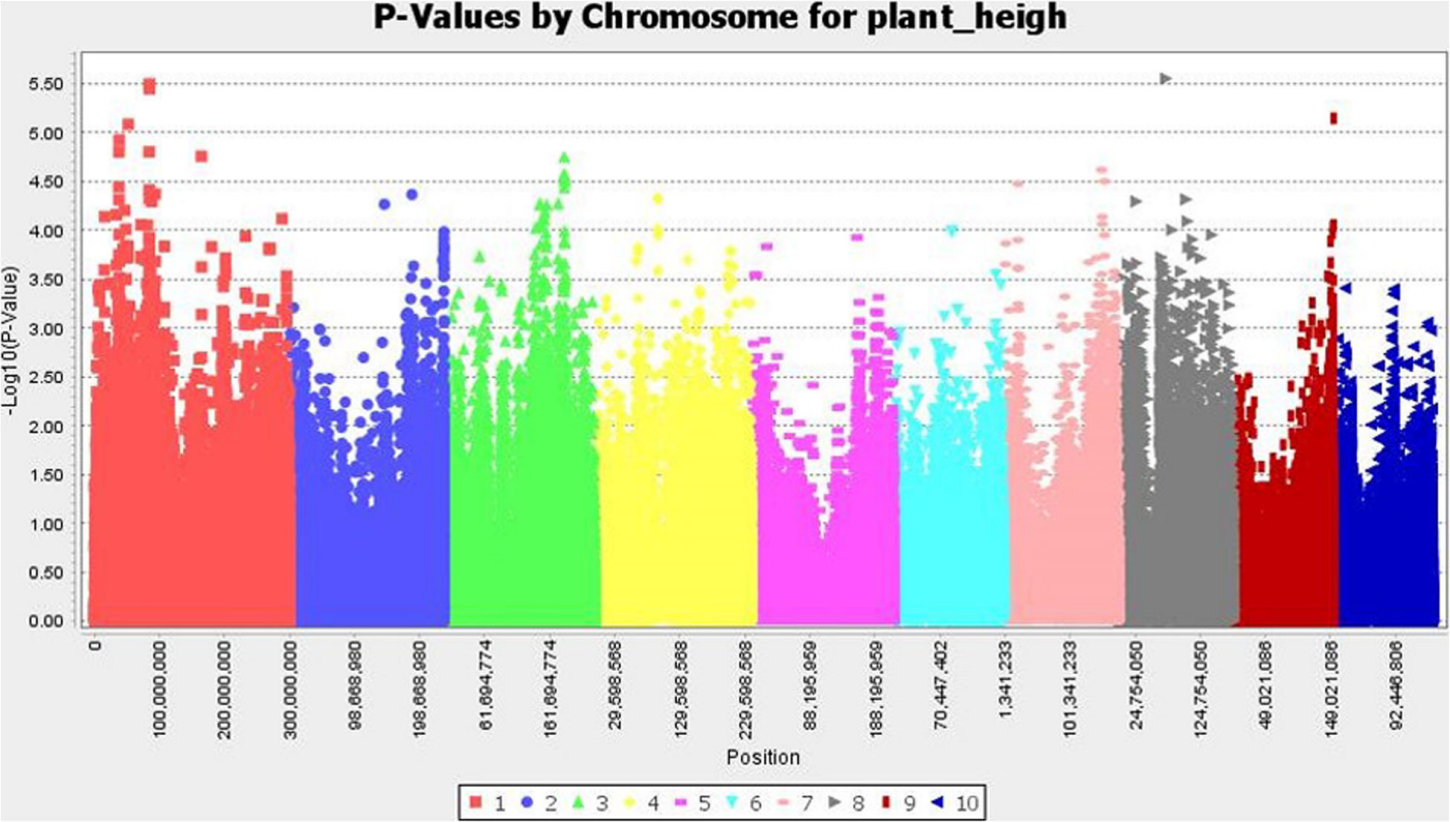
Association between SNPs and plant height. The significance threshold based on deviation of F observed from expected (p=10^-4^) is shown as a horizontal line

The eight parents of the MAGIC population (Table 4) were crossed in pairs to obtain four single crosses that were crossed to obtain two double crosses that were crossed in turn to get a one eight-way cross, described in Butrón et al. [88] (Fig. 7). The eight-way cross was random mated for six generations. In each generation a minimum of 50 crosses were made between 100 different individuals. A bulk was made with the same number of kernels form each ear to contribute to the next generation. After 6 cycles of recombination, we self-pollinated the plants during six generations using the single seed descent method and finally obtained 672 highly homozygous lines (recombinant inbred lines, RILs). Each homozygous line derived from a different plant from the random mating population.

**Table 4:** Parental lines of the MAGIC population

Lines	Grain color	Pedigree	Type of grain
EP17^a^	Yellow	A1267 (Unknown location)^e^	Flint
EP43^a^	Yellow	Parderrubias (Atlantic Spain)^e^	Flint
EP53^a^	Yellow	Laro (Atlantic Spain)^e^	Flint
EP86^a^	Yellow	Nostrano dell’Isola (Italy)^e^	Flint
PB130^b^	Yellow	Rojo Vinoso de Aragón (Mediterranean Spain)^e^	Flint
F473^c^	White	Doré de Gomer (France)^e^	Flint
EP125^a^	Yellow	Selection from CO125	Corn Belt
A509^d^	Yellow	A78 × A109	Corn Belt

^a^From Misión Biológica de Galicia (Spain)

^b^From Estacão Agraria de Braga (Portugal)

^c^From Institut National de la Recherche Agronomique (France)

^d^From University of Minnesota (USA)

^e^European landrace

### Experimental design

The 672 RILs of the MAGIC population together with the parents were evaluated in Pontevedra, Spain (42° 24′N, 8° 38′W and 20 m above sea level) in 2014. The experimental design was a single augmented design with 16 blocks. Forty-two non replicated RILs plus the eight parents of the MAGIC population were randomly assigned to each block. In addition, inbred line EP42 was included in each block as a control susceptible to stalk tunneling. After genotyping, 65 out of the 672 RILs were rejected due to their high level of heterozygosity. The remaining 607 RILs of the MAGIC population were evaluated again in Pontevedra in 2015 using two augmented designs, replicated in the same environment. The experiments were planted manually and each experimental plot consisted of a single row with 17 two-kernel hills, spacing between consecutive hills in a row being 0.18 m and 0.8 m between rows. Plants were thinned after emergence to obtain a final density of ~ 70,000 plants ha^− 1^. Standard agronomical practices were carried out.

The evaluations were made under artificial infestation with eggs of MCB. The eggs for the inoculations were obtained at the Misión Biológica de Galicia by rearing the insects following [89, 90]. Before flowering, a mass of approximately 40 MCB eggs were placed between the stem and the sheath of a basal leaf in five plants per experimental plot.

### Phenotypic data

At harvest the stalks of the infested plants were dissected and the tunnel length (TL) produced by the MCB larvae measured. In each plot, the kernel resistance (KR) was taken on the ears of the infested plants using a subjective visual scale from 1 (totally damaged) to 9 (completely healthy). The following agronomic data were also collected: days to silking (S), plant height (PH), and grain yield (Y). S was measured as the days that elapsed from sowing until 50% of the plants of the plot had silks. PH was taken from the ground to the upper end of the male inflorescence in five representative plants of each plot. Grain yield (Y) was taken using all plants of the plot and expressed as g plant^− 1^ at 140 g H_2_O kg^− 1^. The phenotypic data of EP17 were discarded for the analysis because its phenotype in the field experiments diverged from the typical type expected from this inbred line.

### Genotypic data

The 672 RILs of the MAGIC population and the parents were genotyped for 955,690 SNPs using the genotyping by sequencing (GBS) methodology [91] at the Institute of Biotechnology of the Cornell University. The version 2 of the Maize B73 RefGen was used for locating the markers [92]. The genotyping data were filtered selecting the SNPs sequenced in at least 50% of the RILs and with an allelic frequency higher than 0.05 for the minor allele. In addition, SNPs with more than two alleles and deletions/insertions [93] were eliminated. RILs that were heterozygous for more than 5% of the SNPs were also eliminated. After filtration, the total number of SNPs was 224,363 and the total number of RILs was 607. A neighbor-joining (NJ) tree based on the pairwise similarity coefficients was constructed and a principal component analysis (PCA) was carried out with the program Tassel 5.0 [94] to evaluate the structure of the MAGIC population.

### Statistical analysis of phenotypic data

The best linear unbiased estimator (BLUE) was estimated for each RIL and parent in each environment and across environments using the PROC MIXED of SAS (SAS 9.4, SAS Institute 2016). Environments, repetitions, and blocks were considered as random factors and lines as a fixed effect. Heritabilities (*h*^2^) and correlations among traits were estimated following [48, 95], respectively. The comparison of means was carried out using the Fisher’s protected least significant difference (LSD).

### Association mapping

A genome wide association analysis (GWAS) was performed with the Tassel 5.0 software that uses a mixed linear model (MLM) [94]. Summarizing, the model was:

$$ \mathrm{y}=\mathbf{X}\upbeta +\mathbf{Z}\mathrm{u}+\mathrm{e} $$

where *y* is the vector of BLUEs of the RILs, *β* and u are vectors of the fixed and random effects, respectively, and **X** and **Z** are design matrices. The variances of random effects were modeled as:

$$ Var\ (u)=\mathbf{K}{\upsigma^2}_{\mathrm{a}} $$

where K is the matrix of kinship coefficients and σ^2^_a_ is the estimated additive genetic variance [96]. Estimates of restricted maximum likelihood of the components of the variance were obtained using the “compressed MLM” and the “population parameters previously determined” (P3D) methods described by Zhang et al. [97] and included in Tassel. The experiment-wise threshold for a significant association between a trait and a SNP was determined as the point where the observed and expected F test statistics deviated in the Q-Q plot of the model, resulting in *p* = 1 × 10^− 4^ [98]. Linkage blocks were determined using Haploview software with the solid spine method of linkage disequilibrium (“solid spine of LD”) with D’ > 0.20 [99].

## Supplementary information

**Additional file 1: Figure S1.** A Distribution of grain yield values (g plant^− 1^) in the RILs of the MAGIC population. B Distribution of plant height (cm) in the RILs of the MAGIC population. C Distribution of silking (days from sowing to silking) in the RILs of the MAGIC population.

**Additional file 2: Figure S2.** Neighbor joining cladogram (NJ) of the MAGIC population and their parents.

**Additional file 3: Table S1.** A SNPs significantly associated with silking (days from sowing to silking). The significance of the association, and the variance explained by each SNP are included in the table. B SNPs significantly associated with grain yield (g plant^− 1^). The significance of the association, and the variance explained by each SNP are included in the table. C SNPs significantly associated with plant height (cm). The significance of the association, and the variance explained by each SNP are included in the table.

## Data Availability

The data sets generated and analyzed during the current study and material are available from Rosa Ana Malvar on reasonable request.

## Abbreviations

A: Adenine
Bin: Genome region
BLUE: Best linear umbiased estimator
*Bt*: *Bacillus thuringiensis*
C: Cytosine
DNA: Desoxirribonucleic acid
ECB: European corn borer
G: Guanine
GBS: Genotyping by sequencing
GWAS: Genome wide association analysis
K: Kinship matrix
KR: Kernel resistance
LD: Linkage disequilibrium
LSD: Least significant difference
MAGIC: Multi-parent Advanced Generation InterCrosses
Mb: Mega base pairs
MCB: Mediterranean corn borer
MLM: Mixed linear model
NJ: Neighbor joining
Pb: Base pairs
PCA: Principal component analysis
PH: Plant height
QTL: Quantitative trait loci
RIL: Recombinant inbred lines
S: Silking
SAS: Statistical analysis program
SNP: Single nucleotide polymorphism
T: Thymine
TL: Tunnel length
Y: Yield
