# Supplementary material for: Mapping of resistance to corn borers in a MAGIC population of maize
Source: BMC Plant Biol. 2019 Oct 17;19:431. doi: 10.1186/s12870-019-2052-z (PMC6796440; doi:10.1186/s12870-019-2052-z)

**Figure S1.**

**A Distribution of grain yield values (g plant^-1^) in the RILs of the MAGIC population**


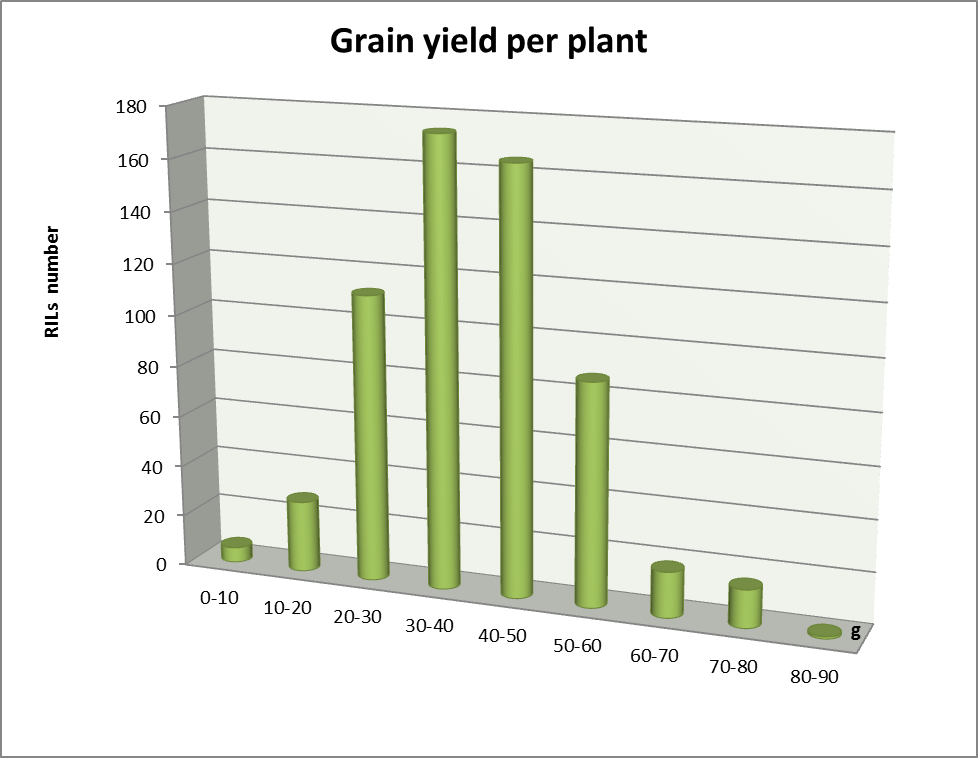


**B. Distribution of plant height (cm) in the RILs of the MAGIC population**


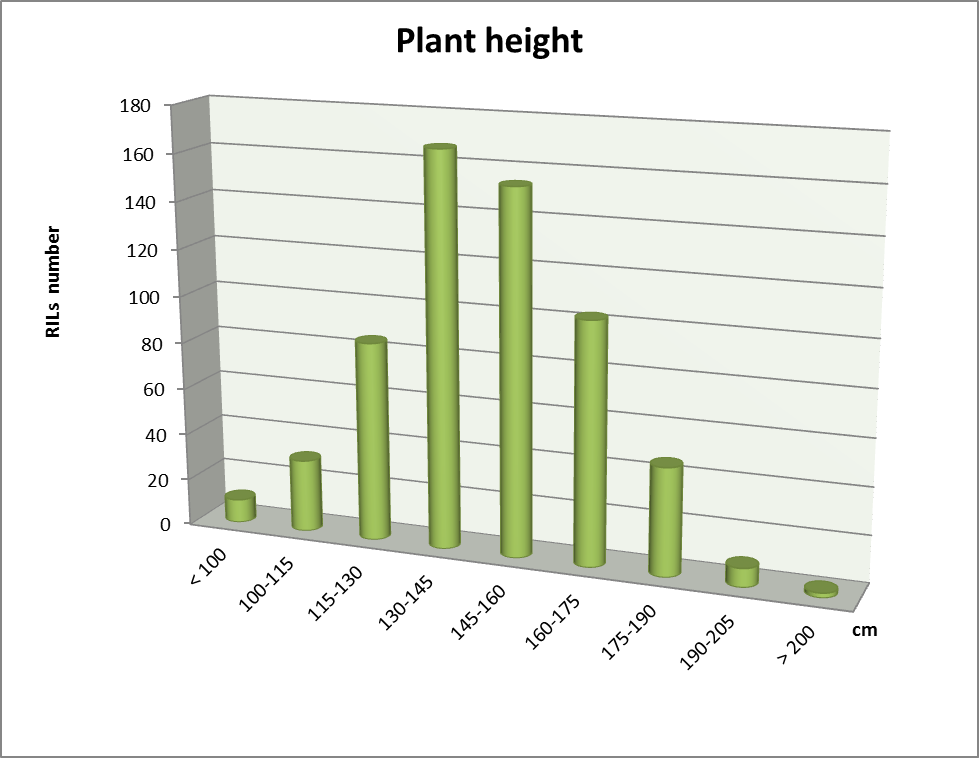


**C Distribution of silking (days from sowing to silking) in the RILs of the MAGIC population**


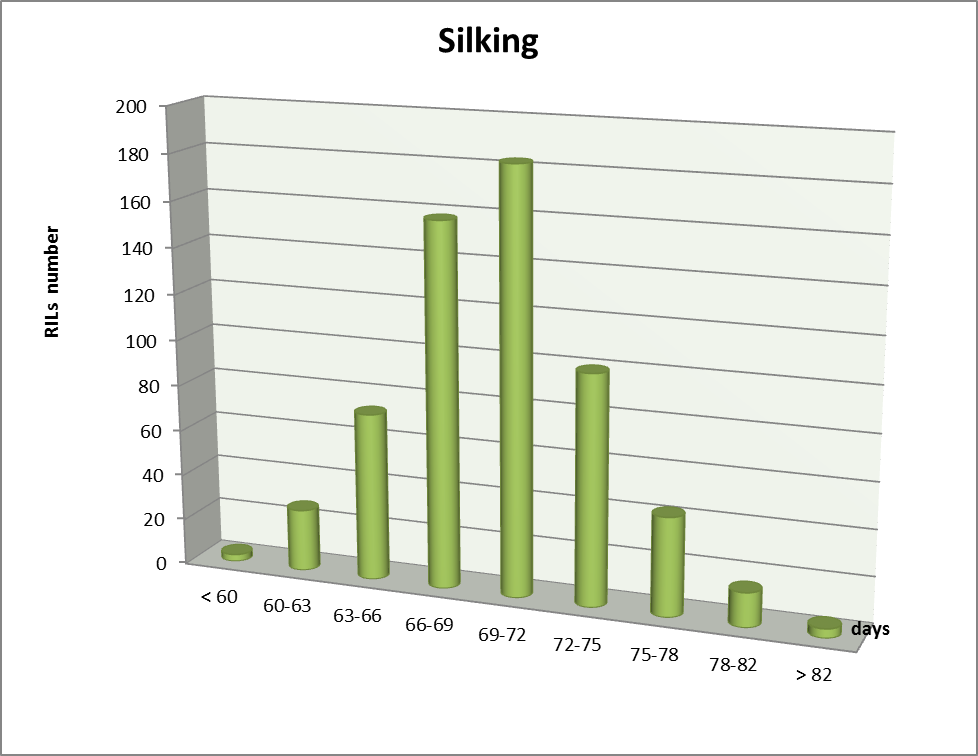

Supplement: Supplementary file 1 — Additional file 1: Figure S1. A Distribution of grain yield values (g plant− 1) in the RILs of the MAGIC population. B Distribution of plant height (cm) in the RILs of the MAGIC population. C Distribution of silking (days from sowing to silking) in the RILs of the MAGIC population. [file 12870_2019_2052_MOESM1_ESM.docx]
