# Supplementary figures and images for: Mapping of resistance to corn borers in a MAGIC population of maize
Source: BMC Plant Biol. 2019 Oct 17;19:431. doi: 10.1186/s12870-019-2052-z (PMC6796440; doi:10.1186/s12870-019-2052-z)

Figure S2. Neighbor joining cladogram (NJ) of the MAGIC population and their parents


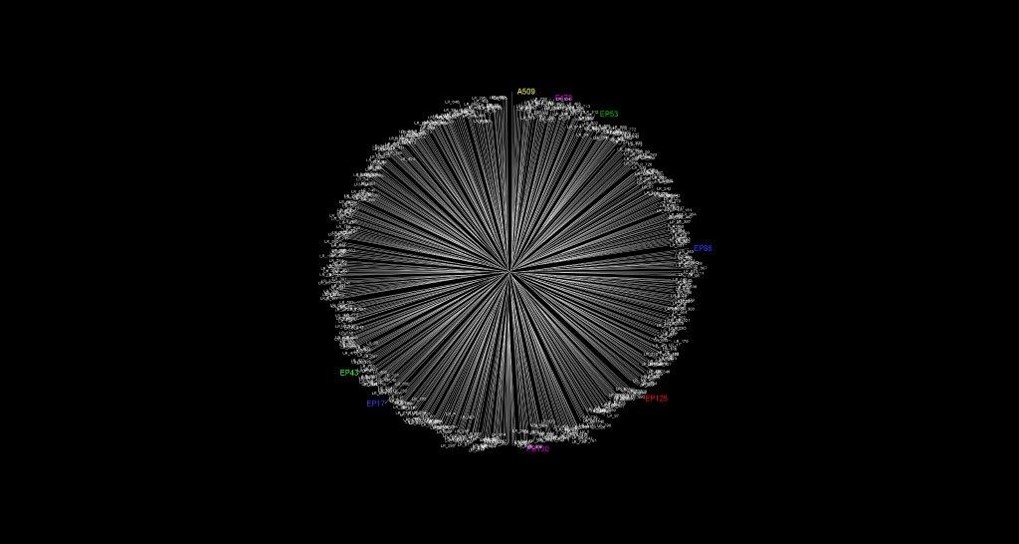

Supplement: Supplementary file 2 — Additional file 2: Figure S2. Neighbor joining cladogram (NJ) of the MAGIC population and their parents. [file 12870_2019_2052_MOESM2_ESM.docx]
